# Patterns and predictors of public dental service utilisation among refugees in Victoria, Australia: a latent profile and multilevel analysis

**DOI:** 10.1186/s12903-023-02886-3

**Published:** 2023-04-04

**Authors:** Prabhakar Veginadu, Mohd Masood, Mark Gussy, Hanny Calache

**Affiliations:** 1 Department of Rural Clinical Sciences, La Trobe Rural Health School, La Trobe University, Bendigo, VIC Australia; 2 Menzies School of Health Research, Alice Springs, Northern Territory, Australia; 3 Dental Institute, University of Turku, Turku, Finland; 4 Lincoln International Institute for Rural Health, University of Lincoln, Brayford Pool, Lincoln, UK

**Keywords:** Refugees, Dental Health Services, Services utilization, Latent class analysis, Multilevel analysis

## Abstract

**Background:**

The purpose of the study was to explore, analyse, and describe the patterns of public dental service utilisation among the refugee populations in Victoria, Australia, and determine their predictors at the individual and contextual levels.

**Methods:**

Data on the refugees who attended Victorian public dental services between July 2016 to June 2020 was gathered from the Dental Health Program dataset. Latent profile analysis was used to identify discrete groups among the refugee clientele with similar mean utilisation patterns across six indicator variables describing the attributes of dental services received and the site of care provision, over the study period. Multilevel multinomial logistic regression analysis was performed to examine the individual and contextual level correlates of the identified utilisation patterns.

**Results:**

Six distinct profiles of public dental service utilisation were identified among the study population (n = 25,542). The largest group comprised refugees predominantly using restorative services under general course of care (38.10%), followed by extraction services under emergency course of care (23.50%). Only a small proportion were estimated as having a higher mean utilisation of preventive services under general course of care (9.10%). Multilevel analysis revealed that the following variables had a significant association with refugee utilisation pattern: at the individual-level – demographic and ethnic attributes including age, gender, region of birth, preferred language for communication, use of language interpreter services, and type of eligibility card; at the contextual-level – characteristics of refugees’ neighbourhood of residence including urbanicity, socioeconomic disadvantage, delivery of Refugee Health Program at the community health centres, and spatial accessibility to public dental services via driving and public transit modes of travel.

**Conclusions:**

The study represents a significant step towards the development of an evidence-based knowledge around public dental service utilisation among Victorian refugees. Overall, the study findings reiterate the critical need for targeted strategies to promote the importance of routine dental visits, oral disease prevention, and timely intervention among refugee groups.

**Supplementary Information:**

The online version contains supplementary material available at 10.1186/s12903-023-02886-3.

## Background

Poor oral health is considered a major burden among the resettled refugee population around the world [1]. This can be attributed to a myriad of past traumatic experiences in their home country, compounded by the challenges related to resettlement in the host country. In Australia, refugees have been shown to demonstrate higher rates of dental caries and periodontal disease compared to the general population [2–4]. This is also acknowledged in the most recent National Oral Health Plan of Australia, 2015–2024 [5], which identifies refugees as a vulnerable group. Timely use of appropriate dental services would contribute to promoting oral health of refugees by providing diagnosis, prevention, and treatment of oral diseases.

In Australia, the Commonwealth government entitles all humanitarian migrants, including refugees, eligibility to access universal healthcare. However, dental services are not included in this. Recognising the vulnerability of the refugee group, the Department of Health and Human Services in the state of Victoria, extended the eligibility for public dental services (PDS) to refugees and asylum seekers [6]. Furthermore, to overcome the frequently noted barriers such as long waiting times and financial constraints, additional policies were introduced to provide refugee populations with ‘priority access,’ where they are accommodated in the next available appointment without being placed on a waiting list, and fee exemption for all services [6]. Despite these measures, data from a 2016 audit suggests limited participation rates among refugees within the Victorian public dental system; approximately 17% of Victorian refugees attended PDS in 2015-16 [7].

Little is known about the characteristics of, and factors associated with dental service utilisation among refugees in Australia, in general, and in Victoria, in particular. Dental service use is considered an important indicator of dental health-related behaviour [8]. Understanding of the pattern of PDS use among the refugee populations provides a valuable insight into their access to dental care; for example, whether their pattern of use comprise routine check-ups and preventive care or is it primarily for the treatment of existing dental problems. In addition, it is also critical to analyse the nature of services received by refugees when visiting a dental service provider (e.g., routine check-up, preventive services or specialist treatment) to identify disparities in the utilisation of particular types of services, investigate problems associated with access to these services, and further examine factors determining their utilisation behaviour. Together, these inform the development of targeted strategies that would enable efficient use of the existing resources by the public dental system to serve this population group.

Previous research examining dental service utilisation behaviour of refugees were primarily conducted using self-reported surveys [9]. Use of survey data in this regard may be limited by the sample size and characteristics, individual’s recollection of past events such as dental attendance [10]. As a result, significant discrepancies were found between self-reported and actual utilisation [11]. Administrative data provides more accurate information on service utilisation, as the treatments received by the patient represent real-life patterns of care and is precisely recorded at the time of care provision [10]. One study used administrative data to investigate public and private dental service utilisation among refugees in Sweden, the results of which showed a low overall use [12]. However, there are no such studies in Australia, warranting research in this space.

This study uses administrative data across a four-year period to examine the utilisation of publicly funded dental services and develop profiles of PDS use among the Victorian refugees. The specific objectives of this study were to: (1) explore patterns of the use of different types of care and services provided through the Victorian public dental health system among the refugee population, (2) analyse and describe the characteristics of refugees with similar patterns of PDS use, (3) examine the association between individual and contextual factors of the refugees and their identified patterns of PDS use.

## Methods

### Data source

This retrospective observational study used secondary data analysis of de-identified individual-level data of refugees who have accessed publicly funded dental services in Victoria. The data was obtained from the electronic dental records sourced from the Victorian Dental Health Program dataset (DHPDS). All data were provided by Dental Health Services Victoria (DHSV).

### Population characteristics

The study population included all refugees, irrespective of their age, gender, or ethnicity, for whom a record was created within the Titanium® patient management system between 01 July 2016 and 30 June 2020. Eligible patients attended the Royal Dental Hospital Melbourne (RDHM) or any of the community dental clinics to avail PDS. Services received were any of the three types of courses of care (CoC) – general, emergency, and denture – and one or more service areas provided as part of any of these three CoCs, including diagnostic, preventive and specialist services.

### Study variables

Measures of dental service utilisation included the number and type of CoCs, services received in each visit during the CoC, and date and clinic of the visit. The data collected from the electronic records contain one record per client per visit during a CoC, which allowed for the measurement of frequency of CoCs and visits per service area per client per year. Individual service items were coded using the coding scheme outlined by the Australian Dental Association [13], and grouped into eleven major service areas – consultations; oral and radiographic examinations; prophylactic and preventive; periodontics; extractions; minor, major and other surgery; endodontics; restorative; crown, bridge and implants; complete and partial dentures; orthodontics. Other variables collected were the type of referral and address (including suburb and postcode) of the clinic site where services were availed.

Individual-level variables collected included client demographics as recorded in their first visit - age, gender, country of birth (stratified according to United Nations geographic regions) [14], preferred language for communication, request for language interpreter service (dummy coded – Yes/No), and type of eligibility card held (health care card, pensioner card, no card).

Context was defined as residential neighbourhood of refugee clients at statistical area level 2 (SA2), corresponding to their residential suburb and postcode collected from the electronic records [15]. SA2s are geographical units defined by the Australian Bureau of Statistics which closely align with the boundaries of suburbs in the metropolitan areas and represent ‘functional communities’ that are socially and economically interactive in outside metropolitan areas [15]. All contextual variables were gathered at SA2 level. These included urbanicity of residence (metropolitan, regional, rural) [16], measure of area-level socioeconomic disadvantage for refugees, whether the Victorian Refugee Health Program (RHP) was delivered at the community health centre (CHC) in the clients’ residential SA2 (dummy coded, Yes/No), and spatial accessibility to PDS via driving and public transit travel modes.

Data on variables indicating refugee socioeconomic disadvantage were obtained from the Australian Census and Migrants Integrated Dataset, 2016 [17]. These included proportion of total resident SA2 population who are refugees and proportion of total refugees in each SA2 who – moved to Australia during the last 5 years (as of 2016), did not complete Year 12, are not proficient in English, are above 15 years and unemployed, have an annual income <$25,999, need assistance with core activities, and live in households without a motor vehicle [18]. Principal Components Analysis was conducted to reduce the dimensionality of these variables and obtain a unified measure of SA2-level socioeconomic disadvantage for the refugee population. Based on the Kaiser’s criterion, five components (capturing 80.02% total variance) with eigenvalues greater than one were combined using their respective eigenvalues as weightings (see Additional file 1 for details). The resulting scores were classified into tertiles. List of RHP sites were gathered from the Victorian Department of Health and were assigned to their respective SA2s based on their suburb and postcode in the postal address [19]. Spatial accessibility to PDS was calculated using the enhanced two-step floating catchment area method individually via road network (for driving mode including car or other motor vehicle) and public transit network (for various public transit modes including bus, tram, metro, or train), as detailed elsewhere [20]. The spatial accessibility index scores obtained from these calculations represent the ratio of full-time equivalent dental professionals to the population eligible for PDS within each SA2, weighted by the travel time between their respective locations via driving or public transit mode; these scores were used as continuous variables.

### Statistical analysis

Latent profile analysis (LPA) was used to identify distinct subgroups that characterise the utilisation of PDS among the Victorian refugee population for the period 2016-17 to 2019-20. LPA is a person-centered model-based approach to identifying underlying subgroups (called latent profiles) based on an unobservable attribute (called latent variable) by assessing multiple dimensions indicated by measured variables (called indicator variables) pertaining to this attribute [21]. The indicator variables can have a continuous, count, or a combination of these distributions. Individuals are probabilistically assigned to the latent profiles based on two model parameters estimated on a maximum-likelihood basis [21]: (a) profile membership probabilities; (b) means and variances of indicator variables, conditional on profile membership. Profiles of individuals sharing similar patterns of the means and variances of each indicator variable are identified and grouped. This enables the distinctness of each identified profiles to be assessed and qualitatively described.

Indicator variables of refugees’ PDS utilisation used in the LPA included attributes of dental services received and clinic sites of service provision, during the four-year period. Number of CoCs received per client in each of the three types of care (i.e., general, emergency, and denture) and by each of two referral types (i.e., self-referral or referred by dental professionals, health care professionals, refugee and community support services, and other support services), as well as number of unique visits in each of the eleven service areas within a CoC were indicators of dental service characteristics. For clinic characteristics, number of CoCs received based on urbanicity of the clinic site (i.e., metropolitan, regional, or rural site) [16], whether the site was within the SA2 of clients’ residence (i.e., within or outside SA2 of residence), and whether the clinic was co-located with CHCs delivering RHP (i.e., co-located or not co-located) were used as indicators. All indicators were counts; hence, a Poisson model was used in the LPA [21, 22]. As there were differences in the number of years in which each client attended PDS, during the study period, it was included as an offset in the Poisson model. Doing so adjusted for any difference in utilisation among the clients by modelling counts as rates (i.e., number of CoCs per year or number of visits per year). Therefore, based on the indicators included in the LPA, the final profile assignment of individuals was based on the combined patterns in the conditional mean rates of utilisation in each of the indicator variables.

LPA model building process was done iteratively. First, a model with one latent profile was fitted. Next, the number of profiles were augmented in a step wise manner until the models no longer converged [23]. From thus obtained models, the best fitted model with the optimal number of profiles was selected based on the following criteria [23, 24]: (a) relative fit statistics – Akaike Information Criterion (AIC) and Bayesian Information Criterion (BIC); (b) classification diagnostics – entropy values closer to 1, average posterior probability of profile membership > 0.7, odds of correct classification based on posterior probabilities > 5 for each profile group; (c) substantive model interpretability and parsimony. Finally, individuals were assigned to a profile based on their maximum posterior probabilities.

Multilevel multinomial logistic regression was performed to examine the role of individual and contextual level variables pertaining to refugees (independent variables) in predicting their profile membership (dependent variable). As clients in the DHPDS data are clustered at the neighbourhood (SA2) level, a two-level random-intercept model was fitted with individuals (level 1) nested within contexts (level 2) [25]. Associations between the independent variables and the profile membership were tested in bivariate analysis, and only variables with significant association (p < .05) were included in the multilevel multivariate analysis. The first model was a null model, which only included the dependent variable with its variance split in the two levels of analysis. Subsequently, individual and contextual level variables were included in blocks in the second and third models, respectively. The association between profile membership and each independent variable was adjusted for in terms of all the other variables included in the models. The amount of contextual-level variation in the patterns of PDS utilisation was determined by estimating intra-class correlation coefficient (ICC) using the null model and the extent to which the independent variables were able to explain this variation was determined by calculating proportional change in variance for Models 2 and 3, in reference to the null model [25]. Coefficients from the regression models were exponentiated to obtain conditional odds ratio (COR) with 95% confidence intervals (CI) [26].

Characteristics of the patients and the identified latent profiles were summarised using frequency and percentages. Chi-squared test was used to examine the differences in distributions of individual and contextual characteristics across the identified profiles. All models were estimated via a Generalised Structural Equation Modelling in Stata 17, using *gsem* command with poisson log link (LPA models) and multinomial logit link (multilevel models) functions [27]. Less than 1.5% of data values were missing on explanatory variables (see Additional file 1, Table S3), which may be considered insignificant relative to the general standard of 5% [28]. So, listwise deletion approach was used to handle missing data. A p value of < 0.05 was considered statistically significant.

## Results

### Descriptive statistics

A total of 25,542 refugee clients attended PDS during the study period receiving a total of 47,919 CoCs, including 31,469 general, 14,887 emergency, and 1563 denture CoCs. In total, clients had 246,119 unique visits across the eleven major service areas. Most of the CoCs were self-referred (88.37%) and were received at a metropolitan clinic (90.62%) located outside clients’ residential SA2 (67.29%). Mean age at the time of first visit was 29.46 years (± 18.15), and females comprised 51.83% of the clientele. Majority were born in countries in the Middle East and North Africa region (38.98%), preferred to communicate in Arabic (28.02%), and resided in the metropolitan region (90.96%).

### LPA model selection

Model selection was based on the statistical fit and the substantive capacity of the model to distinguish between individual PDS use patterns. The model fit was shown to improve with each additional profile; AIC and BIC values continuously decreased as the number of profiles estimated increased (see Additional file 2, Table S4). However, there was only a small improvement in the BIC values (as indicated by ΔBIC) after the six-profile model and signs of model overfitting (with smallest profile comprising < 5% total sample) [29]. Therefore, the six-profile model was selected as the optimal model. The model also adequately differentiates the profiles, as indicated by the entropy value (0.86), high average posterior probability (> 0.8 for each group) and odds of correct classification (> 10 for each group) (see Additional file 2, Table S5).

**Fig. 1:**
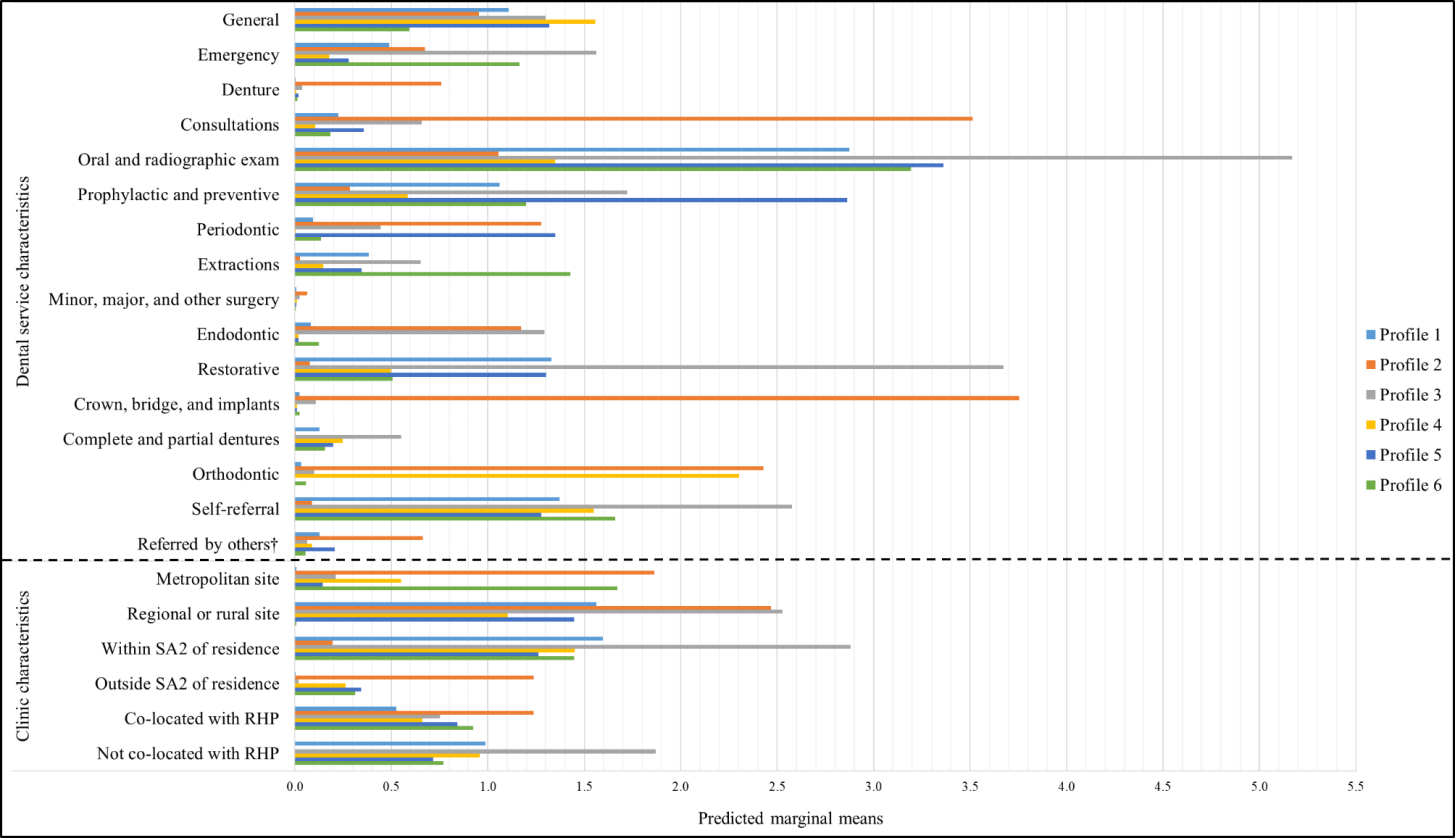
Predicted marginal means of indicators of public dental service use across the six identified profiles. (^†^Others include health care professionals, dental professionals, refugee or community support services, family violence or support services, housing or homelessness services, and educational institutions; Profile 1, ‘General – Restorative’; Profile 2, ‘Denture – Complete and partial dentures’; Profile 3, ‘Emergency – Operative’; Profile 4, ‘General – Orthodontic’; Profile 5, ‘General – Preventive’; Profile 6, ‘Emergency – Extractions’; SA2, statistical area level 2; RHP, refugee health program)

### Profiles of refugee public dental services utilisation

Figure 1 shows the pattern of mean rates of utilisation in each of the indicators across the six identified profiles (see Additional file 2, Table S6). Based on this, descriptors were used to characterise the PDS utilisation pattern of refugees in each group. The profiles were significantly different with respect to the characteristics of refugees assigned to them, as demonstrated in Table 1.

**Table 1:** Individual and contextual characteristics of refugees in the identified profiles

Profiles of PDS use	Overall sample N (%)	Profile 1 n (%)	Profile 2 n (%)	Profile 3 n (%)	Profile 4 n (%)	Profile 5 n (%)	Profile 6 n (%)	p value^†^
**Profile size**	25542 (100)	9732 (38.10)	1619 (6.34)	2189 (8.57)	3671 (14.37)	2324 (9.10)	6007 (23.52)	
**Age (Years)**								< 0.001
0–5	1413 (5.53)	485 (4.98)	7 (0.43)	49 (2.24)	575 (15.66)	33 (1.42)	264 (4.39)	
6–15	5463 (21.39)	1704 (17.51)	35 (2.16)	195 (8.91)	2477 (67.47)	113 (4.86)	939 (15.63)	
16–30	6873 (26.91)	3014 (30.97)	159 (9.82)	636 (29.05)	557 (15.17)	671 (28.87)	1836 (30.56)	
31–45	6593 (25.81)	2674 (27.48)	365 (22.54)	836 (38.19)	38 (1.04)	896 (38.55)	1784 (29.7)	
46–60	3555 (13.92)	1301 (13.37)	529 (32.67)	367 (16.77)	14 (0.38)	478 (20.57)	866 (14.42)	
Above 60	1645 (6.44)	554 (5.69)	524 (32.37)	106 (4.84)	10 (0.27)	133 (5.72)	318 (5.29)	
**Sex**								< 0.05
Male	12066 (47.24)	4499 (46.63)	801 (49.66)	1072 (49.2)	1750 (48.56)	1139 (49.56)	2805 (47.04)	
Female	13239 (51.83)	5149 (53.37)	812 (50.34)	1107 (50.8)	1854 (51.44)	1159 (50.44)	3158 (52.96)	
**Region of birth**								< 0.001
East Asia & Pacific	7163 (28.04)	2629 (27.5)	420 (26.53)	281 (12.99)	1591 (43.84)	857 (37.42)	1385 (23.2)	
Europe, Central Asia, Americas, and Caribbean	157 (0.61)	66 (0.69)	21 (1.33)	19 (0.88)	9 (0.25)	10 (0.44)	32 (0.54)	
Middle East & North Africa	9956 (38.98)	4311 (45.09)	657 (41.5)	1257 (58.11)	1027 (28.3)	552 (24.1)	2152 (36.04)	
South Asia	4404 (17.24)	1380 (14.43)	314 (19.84)	395 (18.26)	610 (16.81)	287 (12.53)	1418 (23.75)	
Sub-Saharan Africa	3517 (13.77)	1175 (12.29)	171 (10.8)	211 (9.75)	392 (10.8)	584 (25.5)	984 (16.48)	
**Preferred language**								< 0.001
English	4291 (16.8)	1693 (17.44)	194 (12)	451 (20.61)	441 (12.06)	487 (20.97)	1025 (17.17)	
Arabic	7158 (28.02)	2975 (30.65)	485 (30.01)	782 (35.74)	805 (22.01)	404 (17.4)	1707 (28.6)	
Persian and Dari	3372 (13.20)	1211 (12.48)	285 (17.64)	421 (19.24)	439 (12)	147 (6.33)	869 (14.56)	
Karen	2431 (9.52)	692 (7.13)	122 (7.55)	43 (1.97)	721 (19.71)	266 (11.46)	587 (9.83)	
Burmese and Related Languages	2191 (8.58)	902 (9.29)	123 (7.61)	103 (4.71)	413 (11.29)	247 (10.64)	403 (6.75)	
Other Languages	6015 (23.55)	2232 (23)	407 (25.19)	388 (17.73)	839 (22.94)	771 (33.2)	1378 (23.09)	
**Request for interpreter service**								< 0.001
Yes	11458 (44.86)	4285 (44.03)	887 (54.79)	1028 (46.96)	1624 (44.24)	1024 (44.06)	2610 (43.45)	
No	14084 (55.14)	5447 (55.97)	732 (45.21)	1161 (53.04)	2047 (55.76)	1300 (55.94)	3397 (56.55)	
**Type of eligibility card**								< 0.001
No card	4840 (18.95)	2234 (22.96)	148 (9.14)	399 (18.23)	611 (16.64)	335 (14.41)	1113 (18.53)	
Health Care card	15445 (60.47)	5815 (59.75)	804 (49.66)	1297 (59.25)	2426 (66.09)	1438 (61.88)	3665 (61.01)	
Pensioner Concession card	5257 (20.58)	1683 (17.29)	667 (41.2)	493 (22.52)	634 (17.27)	551 (23.71)	1229 (20.46)	
**Urbanicity of residence**								< 0.001
Metropolitan	23233 (90.96)	9671 (99.37)	1512 (93.39)	2160 (98.68)	3121 (85.02)	1768 (76.08)	5001 (83.25)	
Regional	1593 (6.24)	47 (0.48)	80 (4.94)	24 (1.1)	511 (13.92)	503 (21.64)	428 (7.13)	
Rural	716 (2.80)	14 (0.14)	27 (1.67)	5 (0.23)	39 (1.06)	53 (2.28)	578 (9.62)	
**Socioeconomic disadvantage (tertile)**								< 0.001
1 (least disadvantaged)	823 (3.22)	405 (4.16)	77 (4.76)	117 (5.35)	115 (3.14)	66 (2.84)	43 (0.72)	
2	5097 (19.96)	2268 (23.31)	357 (22.05)	552 (25.23)	619 (16.88)	525 (22.63)	776 (12.92)	
3	19612 (76.81)	7058 (72.53)	1185 (73.19)	1519 (69.42)	2933 (79.98)	1729 (74.53)	5188 (86.37)	
**Refugee health program in CHCs**								< 0.001
Yes	21045 (82.39)	7635 (78.45)	1304 (80.54)	1670 (76.29)	3167 (86.27)	1841 (79.22)	5428 (90.36)	
No	4497 (17.61)	2097 (21.55)	315 (19.46)	519 (23.71)	504 (13.73)	483 (20.78)	579 (9.64)	
**Driving SPAI scores (tertile)**								< 0.001
1 (lowest accessibility)	4195 (16.42)	824 (8.47)	240 (14.82)	230 (10.51)	906 (24.68)	551 (23.71)	1444 (24.04)	
2	8627 (33.78)	3603 (37.02)	595 (36.75)	825 (37.69)	1148 (31.27)	833 (35.84)	1623 (27.02)	
3	12720 (49.80)	5305 (54.51)	784 (48.42)	1134 (51.8)	1617 (44.05)	940 (40.45)	2940 (48.94)	
**Public transit SPAI scores (tertile)**								< 0.001
1 (lowest accessibility)	3058 (11.97)	1836 (18.87)	177 (10.93)	336 (15.35)	337 (9.18)	208 (8.95)	164 (2.73)	
2	11719 (45.88)	5271 (54.16)	816 (50.4)	1160 (52.99)	1666 (45.38)	1085 (46.69)	1721 (28.65)	
3	10765 (42.15)	2625 (26.97)	626 (38.67)	693 (31.66)	1668 (45.44)	1031 (44.36)	4122 (68.62)	

^†^Chi-squared test; Profile 1, ‘General – Restorative’; Profile 2, ‘Denture – Complete and partial dentures’; Profile 3, ‘Emergency – Operative’; Profile 4, ‘General – Orthodontic’; Profile 5, ‘General – Preventive’; Profile 6, ‘Emergency – Extractions’; PDS, public dental services; CHC, community health centre; SPAI, spatial accessibility index

Profile 1, ‘General – Restorative’ (n = 9732, 38.10%) (herein restorative users), was the largest in size and characterised by higher utilisation rates of general CoC for restorative services. There was also higher uptake of prophylactic and preventive services in this group compared to other groups (except Profile 3 or 5). Clients had the lowest rate of utilisation at clinics co-located with CHCs delivering RHP, located in the regional and rural areas, outside their SA2 of residence, among the profile groups. Individuals were predominantly females, between the ages of 16 and 30, and lived in metropolitan areas most accessible to PDS via driving and least accessible via public transit modes.

Profile 2, ‘Denture – Complete and partial denture’ (n = 1619, 6.34%) (herein denture users), was the smallest and was distinctly characterised by having the highest utilisation rates of denture CoC among all the groups. Not surprisingly, the visits for complete and partial denture services were the highest and extraction services were relatively higher than other groups, except Profile 6. Services were predominantly received at co-located clinic sites. Individuals were relatively older (> 45 years), with higher proportions of males, pensioner concession card holders, and from countries in the Europe, Central Asia, Americas, and Caribbean region, than other groups.

Profile 3, ‘Emergency – Operative’ (n = 2189, 8.57%) (herein operative users), had the highest mean utilisation rates of emergency CoC for endodontic and restorative services. In comparison to other groups, the services received were by self-referral and at metropolitan clinic sites, outside clients’ SA2 of residence. Individuals comprised a higher proportion of those born in the Middle East and North Africa region, spoke Arabic, Persian, or Dari, and lived in areas with least socioeconomic disadvantage for refugees. Within the group, most individuals were between the ages 16 and 45.

Profile 4, ‘General – Orthodontic’ (n = 3671, 14.37%) (herein orthodontic users), had the highest mean rate of general CoCs than other groups, comprising visits predominantly for orthodontic services. Individuals were younger (0–15 years) compared to other groups. The majority had a health care card, spoke Karen, were born in the East Asia and Pacific countries, lived in areas with lowest accessibility to PDS via driving mode, than other groups.

Profile 5, ‘General – Preventive’ (n = 2324, 9.10%) (herein preventive users), predominantly received general CoCs with highest rates of prophylactic and preventive, as well as periodontic services. This group had lowest rates of service utilisation at metropolitan clinic sites not co-located with RHP delivering CHCs. Unlike other groups, majority were between 31 and 45 years, born in countries in the Sub-Saharan Africa region, preferred to communicate in English and Other languages (which included all languages other than the five predominant ones), and lived in the regional areas.

Profile 6, ‘Emergency – Extractions’ (n = 6007, 23.52%) (herein extractions users), was the second largest group and received higher emergency CoCs primarily for extractions, and at clinics located within the clients’ SA2 of residence. Comparing across the profiles, a higher proportion of individuals in this group were from South Asian countries, did not request interpreter services, and lived in rural areas most accessible via public transit and where RHP was delivered.

### Predictors of profile membership

As profile membership (dependent variable) was a multinomial categorical variable, Profile 5 (i.e., preventive users) was used as the reference category in all regression analyses. This was to enable comparison of preventive users and those with a more treatment-oriented pattern of PDS utilisation, i.e., the estimated CORs represent the odds of using a particular service instead of preventive services [26]. Bivariate analysis showed significant associations between each of the individual and contextual level variables of refugees and their profile membership. (Additional file 2, Table S7) Therefore, all independent variables were included in the subsequent multilevel multivariate analysis.

The fitted models along with the estimated effects and their 95% CIs are presented in Table 2. Model 1 is nested within Model 2, which is in turn nested within Model 3. Comparison of the models showed a significant improvement in the fit, as indicated by the reduced AIC and BIC values.

**Table 2:** Multilevel multinomial logistic model explaining associations between profile membership and individual and contextual predictors

	Model 1 COR [95% CI]					Model 2 COR [95% CI]					Model 3 COR [95% CI]				
Profiles of PDS use (Ref.: Profile 5)	Profile 1	Profile 2	Profile 3	Profile 4	Profile 6	Profile 1	Profile 2	Profile 3	Profile 4	Profile 6	Profile 1	Profile 2	Profile 3	Profile 4	Profile 6
**Individual-level variables**															
Age						0.97*** [0.97, 0.97]	1.04*** [1.04, 1.05]	0.98*** [0.97, 0.98]	0.84*** [0.83, 0.84]	0.97*** [0.97, 0.98]	0.97*** [0.97, 0.97]	1.04*** [1.04, 1.05]	0.98*** [0.98, 0.98]	0.83*** [0.83, 0.84]	0.97*** [0.97, 0.98]
Sex (Ref.: Male)															
Female						1.26*** [1.13, 1.39]	1.03 [0.89, 1.18]	1.14* [1.01, 1.30]	1.25*** [1.10, 1.42]	1.19** [1.07, 1.32]	1.26*** [1.14, 1.40]	1.02 [0.88, 1.17]	1.14* [1.00, 1.30]	1.23** [1.08, 1.39]	1.22*** [1.09, 1.36]
Region of birth (Ref.: East Asia and Pacific)															
Europe, Central Asia, Americas, and Caribbean						2.49* [1.21, 5.14]	1.33 [0.58, 3.05]	4.46*** [1.95, 10.18]	1.04 [0.37, 2.92]	2.93** [1.36, 6.32]	2.44* [1.15, 5.21]	1.38 [0.59, 3.27]	4.01** [1.69, 9.51]	0.84 [0.28, 2.55]	3.81** [1.69, 8.57]
Middle East and North Africa						2.05*** [1.59, 2.64]	1.10 [0.79, 1.53]	4.06*** [2.97, 5.54]	1.23 [0.90, 1.67]	2.10*** [1.60, 2.76]	1.84*** [1.42, 2.38]	1.08 [0.78, 1.50]	3.66*** [2.68, 5.01]	1.18 [0.87, 1.61]	2.26*** [1.71, 2.97]
South Asia						0.91 [0.71, 1.17]	1.14 [0.83, 1.57]	1.76*** [1.29, 2.40]	1.04 [0.78, 1.40]	2.57*** [1.97, 3.34]	1.06 [0.82, 1.37]	1.19 [0.86, 1.65]	2.07*** [1.51, 2.84]	0.99 [0.74, 1.32]	2.06*** [1.57, 2.69]
Sub-Saharan Africa						0.86 [0.69, 1.07]	0.59*** [0.44, 0.79]	1.03 [0.77, 1.37]	0.80 [0.62, 1.04]	1.80*** [1.43, 2.27]	1.01 [0.82, 1.26]	0.56*** [0.42, 0.76]	1.09 [0.81, 1.46]	0.68** [0.52, 0.88]	1.52*** [1.20, 1.92]
Preferred language (Ref.: English)															
Arabic						1.18 [0.96, 1.47]	1.11 [0.83, 1.50]	0.77* [0.60, 0.98]	1.39* [1.06, 1.82]	1.65*** [1.32, 2.07]	1.07 [0.86, 1.32]	1.11 [0.82, 1.49]	0.74* [0.58, 0.95]	1.51** [1.15, 1.99]	1.79*** [1.43, 2.25]
Persian and Dari						1.33* [1.04, 1.71]	2.19*** [1.59, 3.02]	1.33* [1.01, 1.75]	1.55** [1.16, 2.09]	1.40** [1.08, 1.81]	1.29* [1.01, 1.66]	2.18*** [1.58, 3.01]	1.32* [1.00, 1.75]	1.50** [1.11, 2.02]	1.42** [1.09, 1.84]
Karen						1.01 [0.76, 1.34]	0.66* [0.44, 0.98]	0.28*** [0.18, 0.45]	2.31*** [1.67, 3.22]	2.24*** [1.66, 3.03]	1.28 [0.95, 1.73]	0.73 [0.48, 1.09]	0.37*** [0.23, 0.58]	1.67** [1.19, 2.33]	1.68*** [1.24, 2.29]
Burmese and Related Languages						1.20 [0.95, 1.53]	0.88 [0.61, 1.26]	0.70* [0.50, 1.00]	1.15 [0.85, 1.55]	1.42** [1.09, 1.85]	1.07 [0.83, 1.36]	0.87 [0.60, 1.25]	0.64* [0.45, 0.92]	1.20 [0.88, 1.62]	1.62*** [1.23, 2.11]
Other Languages						1.16 [0.98, 1.37]	1.24 [0.97, 1.59]	0.76* [0.62, 0.95]	0.97 [0.78, 1.20]	1.14 [0.96, 1.36]	1.05 [0.89, 1.25]	1.26 [0.98, 1.61]	0.74** [0.59, 0.92]	1.04 [0.84, 1.29]	1.19 [0.99, 1.42]
Request for interpreter service (Ref.: Yes)															
No						1.08 [0.96, 1.22]	0.92 [0.79, 1.08]	0.87 [0.75, 1.01]	1.17* [1.01, 1.34]	1.11 [0.98, 1.25]	1.03 [0.92, 1.16]	0.92 [0.78, 1.07]	0.83* [0.71, 0.96]	1.16* [1.01, 1.34]	1.23** [1.09, 1.39]
Type of eligibility card (Ref.: No card)															
Health Care Card						0.70*** [0.60, 0.81]	1.50*** [1.19, 1.90]	0.97 [0.81, 1.17]	1.44*** [1.21, 1.72]	0.95 [0.81, 1.11]	0.68*** [0.58, 0.79]	1.52*** [1.21, 1.93]	0.99 [0.82, 1.19]	1.55*** [1.30, 1.86]	0.90 [0.77, 1.05]
Pensioner Concession Card						0.90 [0.75, 1.08]	2.12*** [1.63, 2.74]	1.42** [1.14, 1.77]	2.74*** [2.20, 3.42]	1.31** [1.09, 1.58]	0.89 [0.74, 1.08]	2.09*** [1.61, 2.71]	1.44** [1.15, 1.80]	2.61*** [2.09, 3.27]	1.19 [0.99, 1.45]
**Contextual-level variables**															
Urbanicity of residence (Ref.: Metropolitan)															
Regional											0.08*** [0.05, 0.15]	0.64 [0.35, 1.16]	0.19*** [0.10, 0.38]	1.48 [0.84, 2.60]	0.42** [0.24, 0.72]
Rural											0.02*** [0.01, 0.06]	0.34* [0.15, 0.78]	0.05*** [0.02, 0.15]	0.15*** [0.07, 0.34]	1.52* [1.21, 3.24]
Socioeconomic disadvantage (tertile) (Ref.: 1st - least disadvantaged)															
2nd											0.73 [0.45, 1.20]	0.70 [0.40, 1.21]	0.70 [0.42, 1.18]	0.53* [0.31, 0.92]	2.12** [1.20, 3.75]
3rd											0.69 [0.42, 1.13]	0.65 [0.37, 1.13]	0.62 [0.36, 1.04]	0.61 [0.35, 1.04]	2.72*** [1.54, 4.81]
Refugee health program in CHC (Ref.: No)															
Yes											0.68* [0.51, 0.92]	0.96 [0.69, 1.33]	0.65** [0.48, 0.89]	0.97 [0.71, 1.34]	1.99*** [1.46, 2.70]
Driving SPAI scores											0.07*** [0.03, 0.15]	0.37* [0.15, 0.88]	0.12*** [0.05, 0.30]	1.60 [0.75, 3.38]	1.57*** [1.15, 3.27]
Public transit SPAI scores											0.28*** [0.16, 0.47]	0.83 [0.47, 1.48]	0.45** [0.25, 0.80]	1.17 [0.70, 1.95]	1.48*** [1.20, 3.11]
**Random effects**															
Variance of the random intercept (SA2 level)	1.15*** [0.87, 1.51]					1.05*** [0.79, 1.39]					0.86*** [0.64, 1.15]				
ICC	0.259					0.242					0.208				
PCV	(Reference)					23.70%					40.22%				
**Model fit indices**															
Number of observations	25542					24890					24880				
Log-likelihood	-39524.68					-33082					-31010.89				
AIC	79061.37					66316					62243.79				
BIC	79110.25					66933.29					63145.31				

***p < .001, ** p < .01, * p < .05; Profile 1, ‘General – Restorative’; Profile 2, ‘Denture – Complete and partial dentures’; Profile 3, ‘Emergency – Operative’; Profile 4, ‘General – Orthodontic’; Profile 5, ‘General – Preventive’; Profile 6, ‘Emergency – Extractions’; COR, conditional odds ratio; CI, confidence interval; PDS, public dental services; CHC, community health centre; SPAI, spatial accessibility index; SA2, statistical area level 2; ICC, intra-class correlation coefficient; PCV, proportional change in variance; AIC, Akaike Information Criterion; BIC, Bayesian Information Criterion.

In Model 1, only the profile membership of refugees was included, with no predictors. The variance of the random intercept (1.15, p < .001) indicated a statistically significant difference between SA2s in the likelihood of refugees belonging to a particular PDS utilisation profile. ICC estimated from the null model (ICC = 0.259) indicated that 25.9% of the total variation in this likelihood is attributed to the differences between the refugees’ SA2 of residence (Table 2); in other words, 74.1% of the variability was accounted for by the individual differences between refugees and other unknown factors.

Model 2 analysed the effect of all individual-level variables. When controlled for the effects of individual variables, the estimated fixed effects continued to remain statistically significant similar to the null model, with a substantial increase in the relative likelihood for restorative and orthodontic users group (COR, 11.16 and 29.82, respectively). Age was a significant predictor of profile membership; the effect of age was positive for denture users and negative for all other profiles. Females relative to males, had a higher odds of belonging to any of the utilisation profiles (COR range, 1.14–1.26), except denture users group. Refugees born in any region were more likely to use extractions and operative services than preventive services (COR range, 1.76–4.46), compared to those born in East Asia and Pacific. Having an eligibility card was significantly associated with a higher odds of orthodontic and denture services use (COR range, 1.44–2.74), than preventive services. The relative likelihood of belonging to the any utilisation profile was higher for Persian and Dari speakers (COR range, 1.33–2.19), compared to those who prefer English. Orthodontic services users were more likely to request interpreter services (COR range, 1.19–1.27), compared to the reference profile group. Together the individual-level predictors explained 23.70% variation in PDS utilisation patterns between SA2s (Table 2).

In the final model, Model 3, contextual-level variables were added to analyse the combined effect of individual and contextual level variables on profile membership. Most of the individual-level variables from the previous model continued to have a significant effect on profile membership, with only a small change in their effect size (max ΔCOR = 0.88). The relative likelihood of orthodontic services use among the refugees from Sub-Saharan countries (COR, 0.68), operative and extraction services use among those who do not request for interpreter services (COR, 0.83 and 1.23, respectively), compared to their corresponding reference categories, gained significance. Urbanicity of residence was a significant predictor of profile membership. For refugees living in rural areas, the relative likelihood of belonging to extractions group was higher (COR, 1.52; 95% CI 1.21–3.24) and lower for the remaining groups (COR range, 0.02 and 0.34), than those living in metropolitan areas. The odds of using restorative, operative, and extraction services, instead of preventive services, decreased for refugees living in regional areas compared to those in metropolitan areas (COR range, 0.08–0.42). Those in the most socioeconomically disadvantaged tertile had a significantly higher relative likelihood of belonging to extractions group (COR, 2.72; 95% CI 1.54–4.18), than those in the lowest tertile. The effect was insignificant for other groups. Increase in spatial accessibility index scores via driving and public transit modes increased the odds of using extraction services by factors of 1.57 and 1.48, respectively. The estimated proportional change in variance for Model 3 indicated that 40.22% of the variation in the patterns of PDS utilisation between different SA2s was explained by the individual and contextual level predictors (Table 2).

## Discussion

The study investigated the patterns and predictive factors of PDS use among refugees in Victoria, using existing administrative data over a four-year period. There was a significant heterogeneity within the study population in terms of the combined patterns and rates of utilisation of different types of CoCs and eleven major service areas as well as the location attributes of clinic sites where they availed PDS. Six distinct profiles of PDS use were identified, described, and subsequently investigated. Together, the findings of this study further the understanding of access and utilisation of PDS among Victorian refugees.

This study is the first to employ LPA to develop profiles of refugee population based on their dental service utilisation pattern. According to the LPA model, the majority of refugees (about 52%) who attended PDS during the study period had a higher probability of using restorative or orthodontic services as part of general CoC. Another 32% predominantly used emergency CoC for extractions or endodontic procedures. Notably, only a very small proportion of refugees (about 9%) used prophylactic and preventive services. These identified patterns were consistent with previous studies which found a low use of preventive services [9, 30], high use of oral surgery and endodontic services [12], and a high proportion of those seeking emergency dental care [3] among refugee populations. Refugees tend to use dental services only when in severe pain or when self-treatments do not work [31]. Consequently, they have a problem-oriented pattern of dental attendance, wherein services are sought infrequently and primarily for treating dental problems [32]. Evidence suggests that a visiting pattern comprising regular dental check-ups and preventive (or interceptive) care is associated with decrease in the use of emergency dental services [33] and better oral health outcomes [34]. Regrettably, this so-called ‘favourable’ utilisation pattern was observed only among a small proportion of refugees in this study.

Examination of refugee characteristics within and across the groups showed a clear distinction between the profiles. Overall, females and young and middle-aged adults (16–45 years) had the most utilisation among the identified profiles, except denture users group (Table 1). This compared favourably with dental visits among the general populations in Australia [32]. Whereas these findings are encouraging, considering the higher burden of dental disease among refugee males and children compared to those in general population [35], a higher uptake among these groups would have been expected. In refugee families, the dental health-related attitude of parents is critical in determining their children’s utilisation pattern, as they are the decision-makers for their children’s dental care needs [36]. So, the lower use among younger age groups may primarily be attributed to their parents. Interestingly though, there was a very high uptake of orthodontic services among children and adolescents (0–15 years), relative to other services, including preventive services. This finding is also substantiated in a study among Australian refugees which reported that the most frequent oral health concern of refugee children or their parents was cosmetic related [4]. Another reason could be that some refugees (with high orthodontic treatment need) can avail orthodontic treatment at no cost via PDS, as opposed to the very expensive private alternatives. Although use of orthodontic services for cosmetic reasons suggest a considerable improvement in oral health attitudes or service awareness among resettled refugees, the findings highlight the need for strategies to improve uptake of preventive services among the 0–15 years group. High utilisation among female refugees is contrary to the literature. It is generally believed that most refugee families have a male dominant structure in which female health-related decisions are made by males [37]. While the study findings suggest otherwise, the reasons for this could be manifold including individual family circumstances, mix of cultural groups within the sample, and differences in the lengths of stay and levels of assimilation to the Australian culture among the study population.

In addition to describing the profile characteristics, the study also determined the predictors of refugee PDS utilisation pattern. At the individual-level, the primary correlates were age and gender. Ethnicity of refugees, based on their region of birth, had a consistently positive association with PDS utilisation pattern, except for denture and orthodontic services use among Sub-Saharan refugees (Table 2). However, there was a considerable difference in the likelihood across profiles among different ethnic groups. Burden of oral diseases may vary among refugees based on their ethnicity owing to the cultural or religious norms, dietary preferences, oral hygiene practices, oral health related attitude including access to dental care in their home country, and their ability to assimilate to the host country’s culture [35, 38]. To some extent, this might have had a decisive influence on their utilisation pattern. Considering the inclination of refugees to resettle in ethnic clusters [39], other factors that could explain the differences may be related to the cultural and social support available to each of these ethnic groups in their communities of settlement. The relationship between the remaining individual variables and utilisation patterns were mixed across the groups.

The role of refugees’ context in predicting their PDS utilisation pattern was confirmed in the current study. About 26% of the variation in refugees’ PDS utilisation patterns was due to the differences in the characteristics of their place of residence (i.e., SA2). A clear gradient was observed between higher SA2-level socioeconomic disadvantage of refugees and increased likelihood of emergency extraction service use. The association between area-level socioeconomic disadvantage and dental service utilisation pattern among refugees reflects on the importance of contextual-level factors in determining PDS utilisation among refugees. This finding is new and an important one. Refugees living in the rural areas were 52% more likely to use emergency extraction services than their metropolitan and regional counterparts. This effect was evident even after adjusting for socioeconomic disadvantage, physical accessibility to community dental clinics via different travel modes and availability of dental professionals, which are considered primary barriers to access among rural residents in Australia [5]. As such, this finding is particularly significant, as it points to factors associated with higher use of extraction services, beyond those considered in the analysis. Such factors may include, but are not limited to, oral health promotion activities [40], social, cultural, or religious networks disseminating information on dental services [38], and presence of community organisations supporting rural refugees in accessing dental care. With refugee resettlement shifting to rural areas [41], it is critical to reorient the public dental system to address these growing inequalities among rural refugee populations.

The study findings add new knowledge on the association between spatial accessibility to dental services and the pattern of utilisation. The latest Australian National Oral Health Plan emphasises the importance of understanding this relationship in order to improve service delivery for vulnerable population groups (including refugees) [5]. The current study found a significant association between potential spatial accessibility to PDS via driving and public transit modes of travel and the utilisation patterns among refugees. Overall, refugees in SA2s least accessible via any travel mode used PDS less than those in most accessible SA2s, which reflects on the impact of potential accessibility on realised service utilisation. Bivariate associations revealed significantly higher likelihood in the use of extraction services than preventive services, with increase in accessibility via any travel mode. When adjusted for the effects of other variables in the multivariate analysis, these associations remained significant. Clearly, this finding implies that irrespective of the level of opportunity to access services, refugees continue to incline toward attending PDS in a problem-oriented manner.

Together, the individual and contextual level factors explained about 40% of the total difference in the utilisation pattern across SA2s; meaning that the remaining 60% variation is due to other factors not included in this study. One of the most important individual-level factors is subjective or objective oral health need, which was found to significantly impact refugee dental service utilisation [37]. Among others, oral health literacy, length of stay in the host country and cultural assimilation were also shown to be positively associated with the utilisation [9, 12]. As well, factors related to the dental health organisation such as cultural competence and responsiveness of dental and support staff, and appropriateness of care provided have been noted to be potential in determining refugees’ utilisation behaviour [37]. Future research should examine the role of these factors on their utilisation pattern.

### Strengths and limitations

This study was the first to have comprehensively evaluated utilisation of PDS among a large sample of refugees in Victoria using administrative data over multiple years. Homogeneity in refugees’ patterns of PDS utilisation was demonstrated using LPA based on multiple indicators including the attributes of dental care and clinic of care reception. This enabled capturing meaningful variations in the complex interactions among different dimensions of PDS utilisation, rather than relying on any one dimension (e.g., either CoC or service type). Moreover, refugees were classified into profiles based on model-based cut-off thresholds derived from within the data, minimising any classification errors that may arise from using arbitrary cut-offs for grouping (e.g., above or below a mean value) [23]. Furthermore, the role of individual and contextual level predictors of PDS utilisation pattern was analysed using a multilevel design.

There are some limitations, primarily arising from the clinical records data. Refugee clients were identified within the DHPDS based on how these individuals were identified and recorded by the public dental clinic staff in the Titanium® system. Although there are a flexible set of criteria available to them to identify an individual as a refugee [42], there is no one agreed upon definition. As such, there may be inconsistencies across clinics. The variables included in the LPA and multilevel analysis were restricted by the availability and completeness of clinical records data. This precluded the evaluation of some important factors known to impact dental service use. For example, there was a large amount missing data (missing for about 69% clients) for variables indicating the oral health status, such as decayed, missing and filled teeth.

The study results must be interpreted within the context of some methodological limitations. The DHPDS does not capture information on those who do not utilise public dental services. As such, factors influencing non-utilisation of public dental services were not evaluated. Profiles developed through LPA are not exclusive [23], i.e., there might be overlap in the services used by refugees in different groups. The assignment of individuals was based on their highest probability of belonging to a particular utilisation pattern which may have resulted in certain amount of misclassification. Due to the focus of the study and cross-sectional design, some dependency structures in the DHPDS data (for e.g., clustering of clients based on their date/year of treatment visit and clinic site of visit) were disregarded which may have resulted in some bias in the model estimates. Finally, as with any study based on administrative data analysis, the findings cannot be generalised outside the study population, i.e., refugees attending PDS in Victoria.

## Conclusions

This study represents a significant step towards the development of an evidence-based knowledge around PDS utilisation among the refugee population in Victoria. Profiles of refugees with distinct patterns of PDS utilisation were developed. The findings demonstrated that the characteristics of refugees’ place of residence including urbanicity, socioeconomic disadvantage, delivery of RHP, and potential spatial accessibility to PDS determined their utilisation pattern. Where opportunities to access PDS were present, refugees were more likely to use extraction services than preventive services. Overall, the findings reiterate the critical need for targeted strategies to promote the importance of routine dental visits, oral disease prevention, and timely intervention among refugee groups.

## Electronic supplementary material

Below is the link to the electronic supplementary material.

Supplementary Material 1

Supplementary Material 2

## Data Availability

The data that support the findings of this study are available from the Manager, Community and Dental Health, Department of Health, Victoria, but restrictions apply to the availability of these data, which were used under license for the current study, and so are not publicly available. Data are however available from the corresponding author upon reasonable request and with permission of the Department of Health, Victoria, and DHSV.

## Abbreviations

AIC: Akaike Information Criterion
BIC: Bayesian Information Criterion
CHC: Community Health Centre
CI: Confidence Interval
CoC: Course of Care
DHSV: Dental Health Services Victoria
ICC: Intra-class Correlation Coefficient
LPA: Latent Profile Analysis
PDS: Public Dental Services
RDHM: Royal Dental Hospital Melbourne
RHP: Refugee Health Program
COR: Conditional Odds Ratio
SA2: Statistical Area Level 2
