# Supplementary material for: Patterns and predictors of public dental service utilisation among refugees in Victoria, Australia: a latent profile and multilevel analysis
Source: BMC Oral Health. 2023 Apr 4;23:201. doi: 10.1186/s12903-023-02886-3 (PMC10074673; doi:10.1186/s12903-023-02886-3)
Supplement: Supplementary file 1 — Supplementary Material 1 [file 12903_2023_2886_MOESM1_ESM.pdf]

## Supplementary Methods

### Calculation of area-based socioeconomic disadvantage measure for refugees

Principal Components Analysis (PCA) was used to obtain a unified measure of socioeconomic disadvantage of refugees from the eight indicator variables mentioned earlier. PCA was used to eliminate collinearity among these variables and determine the appropriate weightings for the resultant uncorrelated components [1, 2]. Before PCA, all variables were standardised to have a mean of 0 and standard deviation of 1. Sampling adequacy for PCA was confirmed through Kaiser-Meyer-Olkin test (KMO = 0.761) and the strength of correlation between the variables was confirmed using Bartlett's test of sphericity ($\chi^2$ = 460.37; degrees of freedom=28; $p<0.001$) [2]. Varimax rotation technique was used to improve the variable loadings on each component [1]. Based on the Kaiser's criterion, five components with eigenvalues greater than 1 were retained (Table S1) [2].

Table S2 shows the variable loadings on the principal components. Variables related to proportion of refugees to the total population, education, English proficiency, and duration of stay loaded heavily on component one. Component two comprised education, duration of stay and needs-assistance variables. Components three, four and five primarily related to having no motor vehicle, unemployment, and low income. Eigenvalues of each component were used as a weight in calculating the unified socioeconomic disadvantage measure for refugees in SA2. The measure was obtained by multiplying the eigenvalues with the corresponding variable loadings in each component (principal component scores) and then taking the sum across all components [3].

**Table S1.** Eigenvalues from the principal component analysis

| Component | Variance | Difference | Proportion | Cumulative |
|---|---|---|---|---|
| 1 | 2.15122 | 1.01972 | 0.2689 | 0.2689 |
| 2 | 1.1315 | 0.044499 | 0.1414 | 0.4103 |
| 3 | 1.087 | 0.0669901 | 0.1359 | 0.5462 |
| 4 | 1.02001 | 0.0081084 | 0.1275 | 0.6737 |
| 5 | 1.0119 | - | 0.1265 | 0.8002 |

Note: only retained components with Eigenvalues greater than 1 are shown

**Table S2.** Loading of variables on each component

| Variable[a] | Component 1 | Component 2 | Component 3 | Component 4 | Component 5 | Unexplained variance |
|---|---|---|---|---|---|---|
| Refugees to total resident SA2 population | **0.5394** | 0.1345 | -0.0948 | 0.2312 | **-0.4275** | 0.2696 |
| Not completed Year 12 | **0.5571** | **-0.3779** | -0.1248 | 0.0373 | 0.2699 | 0.2352 |
| Not proficient in English | **0.5424** | 0.0632 | 0.0761 | -0.1587 | 0.0785 | 0.2673 |
| Arrived in the last five years | **0.3193** | **0.3365** | **0.3335** | -0.2196 | 0.0177 | 0.3309 |
| Above 15 years and unemployed | 0.0114 | 0.0265 | 0.0606 | **0.9272** | 0.042 | 0.08006 |
| Income <$25999 | 0.038 | 0.0875 | -0.0111 | 0.0729 | **0.8537** | 0.1565 |
| Needs assistance with core activities | -0.0113 | **0.8417** | -0.1033 | 0.0484 | 0.0862 | 0.1698 |
| Living in households with no motor vehicle | -0.031 | -0.0709 | **0.9187** | 0.0669 | -0.0031 | 0.08909 |

[a]all variables calculated as proportion of refugees; Number of observations (n) = 348;
Note: Values in bold indicate the largest loading (>|.3|) of each variable on the components

**Table S3.** Missing data for the variables included in multilevel analysis

| Variables | | No. of clients with missing data (% of total clients, N=25542) |
|---|---|---|
| Individual-level variables | Gender | 237 (0.93%) |
| | Preferred language | 84 (0.33%) |
| | Region of birth | 345 (1.35%) |
| Contextual-level variables | Socioeconomic disadvantage | 10 (0.04%) |

## References


1. Wang F. Principal Components, Factor and Cluster Analyses, and Application in Social Area Analysis. In: Wang F, ed. Quantitative methods and socio-economic applications in GIS. 2nd ed. Boca Raton: CRC Press; 2014.
2. Stata Corp. Stata Multivariate Statistics Reference Manual-Release 17. College Station, TX: StataCorp LLC.; 2021.
3. Antony GM, Rao KV. A composite index to explain variations in poverty, health, nutritional status and standard of living: use of multivariate statistical methods. Public Health. 2007;121(8):578-587.
