# Supplementary material for: Patterns and predictors of public dental service utilisation among refugees in Victoria, Australia: a latent profile and multilevel analysis
Source: BMC Oral Health. 2023 Apr 4;23:201. doi: 10.1186/s12903-023-02886-3 (PMC10074673; doi:10.1186/s12903-023-02886-3)
Supplement: Supplementary file 2 — Supplementary Material 2 [file 12903_2023_2886_MOESM2_ESM.pdf]

Supplementary Results

Table S4. Statistical fit indices for the estimated latent profile models

Model	No. of profile groups	Log likelihood	df	AIC	BIC	ΔBIC	Entropy	Size of the smallest profile
1	1	-552503.3	22	1105051	1105230			
2	2	-527160.7	45	1054411	1054778	50452	0.77	42.28%
3	3	-515083.3	68	1030303	1030857	23921	0.79	17.22%
4	4	-508773.4	91	1017729	1018470	12387	0.82	8.06%
5	5	-502599.3	114	1005427	1006356	12114	0.82	7.73%
6	6	-499792.8	137	999859.6	1000976	5380	0.86	6.68%
7	7	-496547.4	160	993414.8	994718.5	6257.5	0.83	4.54%
8	8	-493536.0	183	987438	988929.1	5789.4	0.77	4.39%
9	9	-489189.8	206	978791.5	980470	8459.1	0.82	3.58%
10	10	-487096.9	229	974651.8	976517.7	3952.3	0.80	1.12%

Note: AIC, Akaike information criterion; BIC, Bayesian information criterion; ΔBIC, difference in Bayesian information criteria values

Table S5. Parameters of classification accuracy for each profile group in the selected six-profile model

Predicted profile assignment	Avg. group post probability	Odds of correct classification
1	0.85	10.60
2	0.92	71.75
3	0.88	76.85
4	0.96	13.18
5	0.82	37.33
6	0.91	34.38

Table S6. Predicted marginal means of the indicator variables in each utilisation profile with 95% confidence intervals

Indicators	Profile 1 Mean [95% CI]	Profile 2 Mean [95% CI]	Profile 3 Mean [95% CI]	Profile 4 Mean [95% CI]	Profile 5 Mean [95% CI]	Profile 6 Mean [95% CI]
**Type of course of care**						
General	1.109 [1.085, 1.133]	0.956 [0.910, 1.001]	1.300 [1.254, 1.347]	1.556 [1.520, 1.593]	1.320 [1.273, 1.367]	0.592 [0.569, 0.615]
Emergency	0.490 [0.471, 0.509]	0.675 [0.636, 0.713]	1.563 [1.508, 1.619]	0.177 [0.164, 0.191]	0.278 [0.246, 0.311]	1.165 [1.136, 1.195]
Denture	0.004 [0.003, 0.006]	1.040 [0.989, 1.092]	0.039 [0.031, 0.047]	0.007 [0.001, 0.016]	0.018 [0.012, 0.024]	0.014 [0.011, 0.018]
**Major service areas**						
Consultations	0.226 [0.213, 0.239]	0.759 [0.716, 0.803]	0.656 [0.620, 0.693]	0.106 [0.095, 0.116]	0.359 [0.324, 0.393]	0.184 [0.171, 0.196]
Oral and radiographic exam	2.874 [2.832, 2.916]	3.514 [3.420, 3.608]	5.170 [5.059, 5.281]	1.350 [1.311, 1.389]	3.362 [3.265, 3.458]	3.194 [3.142, 3.246]
Prophylactic and preventive	1.061 [1.030, 1.093]	1.056 [1.004, 1.108]	1.721 [1.660, 1.783]	0.585 [0.561, 0.609]	2.863 [2.766, 2.960]	1.198 [1.165, 1.231]
Periodontic	0.094 [0.084, 0.105]	0.285 [0.257, 0.313]	0.443 [0.412, 0.475]	0.005 [0.003, 0.007]	1.350 [1.270, 1.428]	0.136 [0.124, 0.149]
Extractions	0.382 [0.368, 0.398]	1.277 [1.221, 1.332]	0.653 [0.616, 0.689]	0.147 [0.135, 0.160]	0.347 [0.316, 0.377]	1.428 [1.409, 1.447]
Minor, major, and other surgery	0.008 [0.006, 0.010]	0.028 [0.020, 0.036]	0.024 [0.018, 0.031]	0.011 [0.008, 0.020]	0.007 [0.003, 0.011]	0.004 [0.002, 0.006]
Endodontic	0.082 [0.074, 0.091]	0.062 [0.049, 0.076]	1.293 [1.240, 1.346]	0.018 [0.014, 0.022]	0.017 [0.010, 0.025]	0.124 [0.112, 0.135]
Restorative	1.330 [1.295, 1.365]	1.172 [1.114, 1.230]	3.672 [3.584, 3.761]	0.498 [0.475, 0.522]	1.302 [1.228, 1.377]	0.507 [0.469, 0.545]
Crown, bridge, and implants	0.023 [0.019, 0.027]	0.078 [0.064, 0.092]	0.107 [0.093, 0.121]	0.010 [0.002, 0.019]	0.010 [0.004, 0.016]	0.025 [0.020, 0.029]
Complete and partial dentures	0.127 [0.117, 0.138]	3.754 [3.650, 3.858]	0.552 [0.516, 0.588]	0.248 [0.232, 0.264]	0.199 [0.172, 0.225]	0.155 [0.142, 0.168]
Orthodontic	0.033 [0.027, 0.039]	0.005 [0.000, 0.009]	0.099 [0.083, 0.115]	2.302 [2.249, 2.357]	0.003 [0.001, 0.005]	0.059 [0.049, 0.070]

**Referral**						
Self-referral	1.373 [1.344, 1.401]	2.429 [2.355, 2.504]	2.576 [2.507, 2.646]	1.550 [1.513, 1.587]	1.276 [1.220, 1.332]	1.660 [1.623, 1.696]
Referred by others†	0.128 [0.118, 0.137]	0.090 [0.075, 0.104]	0.063 [0.051, 0.074]	0.088 [0.079, 0.098]	0.206 [0.183, 0.229]	0.055 [0.048, 0.062]
**Location of clinic**						
Within SA2 of residence	0.007 [0.002, 0.015]	0.662 [0.618, 0.707]	0.213 [0.182, 0.243]	0.550 [0.525, 0.575]	0.143 [0.119, 0.167]	1.672 [1.636, 1.708]
Outside SA2 of residence	1.564 [1.535, 1.592]	1.862 [1.795, 1.929]	2.527 [2.458, 2.596]	1.104 [1.072, 1.137]	1.448 [1.385, 1.510]	0.008 [0.004, 0.010]
**Urbanicity of clinic**						
Metropolitan	1.596 [1.566, 1.626]	2.467 [2.392, 2.542]	2.880 [2.807, 2.953]	1.449 [1.413, 1.485]	1.264 [1.211, 1.317]	1.449 [1.414, 1.483]
Regional and rural	0.006 [0.001, 0.012]	0.196 [0.171, 0.221]	0.020 [0.010, 0.029]	0.261 [0.245, 0.278]	0.344 [0.312, 0.377]	0.312 [0.296, 0.329]
**Co-location of clinic**						
Co-located with RHP	0.527 [0.507, 0.547]	1.238 [1.180, 1.296]	0.754 [0.708, 0.799]	0.662 [0.636, 0.687]	0.843 [0.790, 0.897]	0.925 [0.897, 0.952]
Not co-located with RHP	0.988 [0.963, 1.014]	1.237 [1.179, 1.293]	1.870 [1.807, 1.934]	0.956 [0.926, 0.986]	0.715 [0.670, 0.760]	0.770 [0.745, 0.795]

†Others include health care professionals, dental professionals, refugee or community support services, family violence or support services, housing or homelessness services, and educational institutions; Profile 1, 'General – Restorative'; Profile 2, 'Denture – Complete and partial dentures'; Profile 3, 'Emergency – Operative'; Profile 4, 'General – Orthodontic'; Profile 5, 'General – Preventive'; Profile 6, 'Emergency – Extractions'; SA2, statistical area level 2; RHP, refugee health program.

Table S7. Bivariate and multilevel multivariate analyses results

	Unadjusted model cCOR [95% CI]						Fully adjusted model aCOR [95% CI]				
Profiles of public dental service use (Ref.: Profile 5)	**Profile 1**	**Profile 2**	**Profile 3**	**Profile 4**	**Profile 6**	**χ2 (df)**	**Profile 1**	**Profile 2**	**Profile 3**	**Profile 4**	**Profile 6**
**Individual-level variables**											
**Age**	0.98*** [0.97, 0.98]	1.05*** [1.04, 1.05]	0.99*** [0.99, 1.00]	0.85*** [0.84, 0.85]	0.98*** [0.97, 0.98]	9137.47 (5)***	0.97*** [0.97, 0.97]	1.04*** [1.04, 1.05]	0.98*** [0.98, 0.98]	0.83*** [0.83, 0.84]	0.97*** [0.97, 0.98]
**Sex**	(Ref.: Male)					14.15 (5)*					
Female	1.12* [1.03, 1.23]	1.00 [0.88, 1.13]	1.01 [0.90, 1.14]	1.04 [0.94, 1.16]	1.11* [1.01, 1.22]		1.26*** [1.14, 1.40]	1.02 [0.88, 1.17]	1.14* [1.00, 1.30]	1.23** [1.08, 1.39]	1.22*** [1.09, 1.36]
**Region of birth**	(Ref.: East Asia & Pacific)					1692.30 (20)***					
Europe, Central Asia, Americas, and Caribbean	2.15* [1.10, 4.20]	4.29*** [2.00, 9.18]	5.79*** [2.66, 12.61]	0.48 [0.20, 1.20]	1.98 [0.97, 4.05]		2.44* [1.15, 5.21]	1.38 [0.59, 3.27]	4.01** [1.69, 9.51]	0.84 [0.28, 2.55]	3.81** [1.69, 8.57]
Middle East & North Africa	2.55*** [2.26, 2.86]	2.43*** [2.06, 2.86]	6.94*** [5.87, 8.21]	1.10 [0.88, 1.14]	2.41*** [2.13, 2.74]		1.84*** [1.42, 2.38]	1.08 [0.78, 1.50]	3.66*** [2.68, 5.01]	1.18 [0.87, 1.61]	2.26*** [1.71, 2.97]
South Asia	1.57*** [1.35, 1.82]	2.23*** [1.83, 2.72]	4.20*** [3.43, 5.14]	1.14 [0.97, 1.35]	3.06*** [2.62, 3.56]		1.06 [0.82, 1.37]	1.19 [0.86, 1.65]	2.07*** [1.51, 2.84]	0.99 [0.74, 1.32]	2.06*** [1.57, 2.69]
Sub-Saharan Africa	0.66*** [0.58, 0.74]	0.60*** [0.49, 0.73]	1.10 [0.90, 1.36]	0.36*** [0.31, 0.42]	1.04 [0.91, 1.19]		1.01 [0.82, 1.26]	0.56*** [0.42, 0.76]	1.09 [0.81, 1.46]	0.68** [0.52, 0.88]	1.52*** [1.20, 1.92]
**Preferred language**	(Ref.: English)					1359.91 (25)***					
Arabic	2.12*** [1.83, 2.45]	3.01*** [2.44, 3.73]	2.09*** [1.75, 2.49]	2.20*** [1.85, 2.62]	2.01*** [1.72, 2.34]		1.07 [0.86, 1.32]	1.11 [0.82, 1.49]	0.74* [0.58, 0.95]	1.51** [1.15, 1.99]	1.79*** [1.43, 2.25]

Persian and Dari	2.37***<br>[1.94, 2.89]	4.87***<br>[3.75, 6.31]	3.09***<br>[2.46, 3.88]	3.30***<br>[2.63, 4.14]	2.81***<br>[2.29, 3.45]		1.29*<br>[1.01, 1.66]	2.18***<br>[1.58, 3.01]	1.32*<br>[1.00, 1.75]	1.50**<br>[1.11, 2.02]	1.42**<br>[1.09, 1.84]
Karen	0.75**<br>[0.63, 0.89]	1.15<br>[0.88, 1.51]	0.17***<br>[0.12, 0.25]	2.99***<br>[2.47, 3.62]	1.05<br>[0.88, 1.26]		1.28<br>[0.95, 1.73]	0.73<br>[0.48, 1.09]	0.37***<br>[0.23, 0.58]	1.67**<br>[1.19, 2.33]	1.68***<br>[1.24, 2.29]
Burmese and Related Languages	1.05<br>[0.88, 1.25]	1.25<br>[0.95, 1.64]	0.45***<br>[0.35, 0.59]	1.85***<br>[1.51, 2.26]	0.78**<br>[0.64, 0.94]		1.07<br>[0.83, 1.36]	0.87<br>[0.60, 1.25]	0.64*<br>[0.45, 0.92]	1.20<br>[0.88, 1.62]	1.62***<br>[1.23, 2.11]
Other Languages	0.83**<br>[0.73, 0.95]	1.33**<br>[1.08, 1.63]	0.54***<br>[0.46, 0.65]	1.20*<br>[1.02, 1.41]	0.85*<br>[0.74, 0.98]		1.05<br>[0.89, 1.25]	1.26<br>[0.98, 1.61]	0.74**<br>[0.59, 0.92]	1.04<br>[0.84, 1.29]	1.19<br>[0.99, 1.42]
**Request for interpreter service**			(Ref.: Yes)			76.67 (5)***					
No	1.00<br>[0.91, 1.10]	0.65***<br>[0.57, 0.74]	0.89<br>[0.79, 1.00]	0.99<br>[0.89, 1.10]	1.03<br>[0.93, 1.13]		1.03<br>[0.92, 1.16]	0.92<br>[0.78, 1.07]	0.83*<br>[0.71, 0.96]	1.16*<br>[1.01, 1.34]	1.23**<br>[1.09, 1.39]
**Type of eligibility card**			(Ref.: No card)			620.19 (10)***					
Health Care Card	0.61***<br>[0.53, 0.69]	1.27*<br>[1.02, 1.56]	0.76***<br>[0.64, 0.89]	0.92<br>[0.80, 1.07]	0.77***<br>[0.67, 0.88]		0.68***<br>[0.58, 0.79]	1.52***<br>[1.21, 1.93]	0.99<br>[0.82, 1.19]	1.55***<br>[1.30, 1.86]	0.90<br>[0.77, 1.05]
Pensioner Concession Card	0.46***<br>[0.39, 0.53]	2.74***<br>[2.19, 3.43]	0.75**<br>[0.62, 0.91]	0.63***<br>[0.53, 0.75]	0.67***<br>[0.57, 0.79]		0.89<br>[0.74, 1.08]	2.09***<br>[1.61, 2.71]	1.44**<br>[1.15, 1.80]	2.61***<br>[2.09, 3.27]	1.19<br>[0.99, 1.45]
**Contextual-level variables**											
**Urbanicity of residence**			(Ref.: Metropolitan)			3220.30 (10)***					
Regional	0.02***<br>[0.01, 0.03]	0.19***<br>[0.15, 0.24]	0.04***<br>[0.03, 0.06]	0.58***<br>[0.50, 0.66]	0.30***<br>[0.26, 0.35]		0.08***<br>[0.05, 0.15]	0.64<br>[0.35, 1.16]	0.19***<br>[0.10, 0.38]	1.48<br>[0.84, 2.60]	0.42**<br>[0.24, 0.72]

Rural	0.05*** [0.03, 0.09]	0.60* [0.37, 0.95]	0.08*** [0.03, 0.19]	0.42*** [0.27, 0.63]	2.86*** [1.90, 4.13]		0.02*** [0.01, 0.06]	0.34* [0.15, 0.78]	0.05*** [0.02, 0.15]	0.15*** [0.07, 0.34]	1.52* [1.21, 3.24]
**Socioeconomic disadvantage (tertile)**			(Ref.: 1st - least disadvantaged)			630.93 (10)***					
2nd	0.70* [0.53, 0.93]	0.58** [0.41, 0.83]	0.59** [0.43, 0.82]	0.68* [0.49, 0.94]	2.27*** [1.52, 3.38]		0.73 [0.45, 1.20]	0.70 [0.40, 1.21]	0.70 [0.42, 1.18]	0.53* [0.31, 0.92]	2.12** [1.20, 3.75]
3rd	0.67** [0.51, 0.87]	0.59** [0.42, 0.82]	0.50** [0.36, 0.68]	0.97 [0.72, 1.33]	4.61*** [3.12, 6.79]		0.69 [0.42, 1.13]	0.65 [0.37, 1.13]	0.62 [0.36, 1.04]	0.61 [0.35, 1.04]	2.72*** [1.54, 4.81]
**Refugee health program in CHC**			(Ref.: No)			514.76 (5)***					
Yes	0.96 [0.85, 1.07]	1.09 [0.93, 1.27]	0.84* [0.73, 0.97]	1.65*** [1.44, 1.89]	2.46*** [2.16, 2.81]		0.68* [0.51, 0.92]	0.96 [0.69, 1.33]	0.65** [0.48, 0.89]	0.97 [0.71, 1.34]	1.99*** [1.46, 2.70]
**Driving SPAI scores**	0.01*** [0.01, 0.02]	0.24*** [0.15, 0.40]	0.03*** [0.02, 0.05]	1.83*** [1.30, 2.58]	1.72** [1.24, 2.38]	1594.06 (5)***	0.07*** [0.03, 0.15]	0.37* [0.15, 0.88]	0.12*** [0.05, 0.30]	1.60 [0.75, 3.38]	1.57*** [1.15, 3.27]
**Public transit SPAI scores**	0.07*** [0.06, 0.09]	0.40*** [0.31, 0.52]	0.12*** [0.09, 0.17]	1.03 [0.91, 1.18]	1.25*** [1.11, 1.41]	1773.47 (5)***	0.28*** [0.16, 0.47]	0.83 [0.47, 1.48]	0.45** [0.25, 0.80]	1.17 [0.70, 1.95]	1.48*** [1.20, 3.11]

***p<.001, ** p<.01, * p<.05; Profile 1, ‘General – Restorative’; Profile 2, Denture – ‘Complete and partial dentures’; Profile 3, ‘Emergency – Operative’; Profile 4, ‘General – Orthodontic’; Profile 5, ‘General – Preventive’; Profile 6, ‘Emergency – Extractions’; cCOR, crude conditional odds ratio; aCOR, adjusted conditional odds ratio; CI, confidence interval; $\chi2$, Chi-squared test; df, degrees of freedom; CHC, community health centre; SPAI, spatial accessibility index
